# Comparative Genomic and Pan-Genomic Characterization of *Staphylococcus epidermidis* From Different Sources Unveils the Molecular Basis and Potential Biomarkers of Pathogenic Strains

**DOI:** 10.3389/fmicb.2021.770191

**Published:** 2021-11-15

**Authors:** Shudan Lin, Bianjin Sun, Xinrui Shi, Yi Xu, Yunfeng Gu, Xiaobin Gu, Xueli Ma, Tian Wan, Jie Xu, Jianzhong Su, Yongliang Lou, Meiqin Zheng

**Affiliations:** ^1^Zhejiang Provincial Key Laboratory for Technology and Application of Model Organisms, Key Laboratory of Laboratory Medicine, Ministry of Education, School of Laboratory Medicine and Life Sciences, Wenzhou Medical University, Wenzhou, China; ^2^School of Ophthalmology and Optometry and Eye Hospital, School of Biomedical Engineering, Wenzhou Medical University, Wenzhou, China

**Keywords:** *Staphylococcus epidermidis*, ocular, whole genome, pan-genomic, evolution, comparative genomic, virulence gene, antimicrobial resistance

## Abstract

Coagulase-negative *Staphylococcus* (CoNS) is the most common pathogen causing traumatic endophthalmitis. Among which, *Staphylococcus epidermidis* is the most common species that colonizes human skin, eye surfaces, and nasal cavity. It is also the main cause of nosocomial infection, specially foreign body-related bloodstream infections (FBR-BSIs). Although some studies have reported the genome characteristics of *S. epidermidis*, the genome of ocular trauma-sourced *S. epidermidis* strain and a comprehensive understanding of its pathogenicity are still lacking. Our study sequenced, analyzed, and reported the whole genomes of 11 ocular trauma-sourced samples of *S. epidermidis* that caused traumatic endophthalmitis. By integrating publicly available genomes, we obtained a total of 187 *S. epidermidis* samples from healthy and diseased eyes, skin, respiratory tract, and blood. Combined with pan-genome, phylogenetic, and comparative genomic analyses, our study showed that *S. epidermidis*, regardless of niche source, exhibits two founder lineages with different pathogenicity. Moreover, we identified several potential biomarkers associated with the virulence of *S. epidermidis*, including *essD*, *uhpt*, *sdrF*, *sdrG*, *fbe*, and *icaABCDR*. *EssD* and *uhpt* have high homology with *esaD* and *hpt* in *Staphylococcus aureus*, showing that the genomes of *S. epidermidis* and *S. aureus* may have communicated during evolution. *SdrF*, *sdrG*, *fbe*, and *icaABCDR* are related to biofilm formation. Compared to *S. epidermidis* from blood sources, ocular-sourced strains causing intraocular infection had no direct relationship with biofilm formation. In conclusion, this study provided additional data resources for studies on *S. epidermidis* and improved our understanding of the evolution and pathogenicity among strains of different sources.

## Introduction

Coagulase-negative *staphylococci* (CoNS) usually live on human skin (Piette and Verschraegen, 2009), nasal cavity (97%; Wos-Oxley et al., 2010), and ocular surface (60%; Graham et al., 2007; Willcox, 2013) and is the most common isolate recovered from nosocomial bloodstream infections (31%; Wisplinghoff et al., 2004). *Staphylococcus epidermidis* is the most common CoNS species (Otto, 2004), and it plays a central role in the skin microbiota; for example, it can protect against colonization by skin pathogens (Cogen et al., 2010a,b), maintain the ecological balance of human skin flora (Schommer and Gallo, 2013), and modulate the immune system (Egert et al., 2017; Linehan et al., 2018). Once it breaches the skin surface and enters the bloodstream, however, it is considered pathogenic. As the second most common cause of nosocomial infections (Otto, 2009), *S. epidermidis* not only substantially accounts for foreign body-related infections (Becker et al., 2014) but also causes many eye infections (Keay et al., 2006; Schimel et al., 2013; Bispo et al., 2014; Park et al., 2015b). Previous study showed that CoNS, including *S. epidermidis*, was the most common pathogen causing traumatic endophthalmitis accompanied by intraocular foreign body (Al-Omran et al., 2007), and the average visual acuity of an eye is usually at the low level (Bhagat et al., 2011; Cornut et al., 2013). The different roles of *S. epidermidis* in health and disease make it as one of the essential bacterial species of the human microbiota. Although a high positive rate of *S. epidermidis* was detected in clinical specimens, whether they represent actual infection or only colonization/contamination remains to be discussed.

Although much less is known regarding the potential risk of *S. epidermidis* to cause outbreaks, the biofilm formation and antibiotic resistance of *S. epidermidis* contribute to the occurrence and persistence of clinical infections (Schoenfelder et al., 2010), strongly suggesting that modern medicine has facilitated the selection process, mainly by the (over)use of antibiotics and the insertion of foreign body devices (Becker et al., 2014). Detachment of bacterial cells from the biofilm on the medical devices can lead to bacteremia and increase morbidity and potential mortality (Conlan et al., 2012). It is well-known that biofilms are resistant to antibacterial drugs (Conlan et al., 2012). Antibiotic resistance significantly complicates treatment and increases medical costs (Foster, 2017; Lee et al., 2018). The gene *mecA* is present on staphylococcal chromosome cassette *mec* (SCC*mec*) and encodes the penicillin-binding protein PBP2a (Katayama et al., 2000), which may confer resistance to methicillin (Hartman and Tomasz, 1984). Although methicillin is not used to treat eye infections, it is known that increasing resistance to methicillin can significantly promote the spread and persistence of multidrug-resistant strains in specific environments (Alekshun and Levy, 2007; Asbell et al., 2008; Lichtinger et al., 2012).

Recently, with the development of high-throughput sequencing technology, the genome of *S. epidermidis* has been extensively studied, including full pan-genome analysis of both commensal and nosocomial isolates (Conlan et al., 2012), identification of the presence of *S. epidermidis* lineages in healthy individuals from two geographical locations (Sharma et al., 2018), genomic determinants associated with their adaptation to various environments (Su et al., 2020), and the evolutionary trajectory and functional distribution of *S. epidermidis* (Zhou et al., 2020). Microorganisms can be shaped by different host-specific factors, such as disease and health status. However, it remains unclear whether the detected *S. epidermidis* strains were true infectious pathogen or contaminant, especially in the case of respiratory tract samples, which were generally considered as contamination and were not reported. Thus, the pathogenic nature of *S. epidermidis* from various host health conditions should be fully investigated. Moreover, although Kirstahler et al. (2018) reported six whole genomes of *S. epidermidis* from vitreous humor, a comprehensive understanding of the genome of ocular strains, particularly ocular trauma-sourced strains, is still lacking, and the genetic differences among strains isolated from different host niches remained unclear.

In this study, we sequenced the whole genomes of 11 ocular trauma-sourced *S. epidermidis* isolates. Along with incorporating publicly available genomes, we obtained a total of 187 genomic sequences of *S. epidermidis* isolates from eyes, skin, respiratory tract, and blood of healthy and diseased hosts. Through whole-genome sequence (WGS) analysis and pan-genome analysis, we performed a detailed understanding among *S. epidermidis* isolates from diverse host health status and within-individual sources. We comprehensively analyzed and reported the WGS of *S. epidermidis* that causes traumatic endophthalmitis. With phylogenetic analysis and comparative genomics, we revealed the evolutionary relationships of different sources, exploring how different host niches may shape the genetic diversity of *S. epidermidis*. Our study demonstrated a marked association between evolutionary lineage and host health states. Regardless of niche source, *S. epidermidis* showed two founder lineages with different pathogenicity. We also identified several potential biomarkers related to *S. epidermidis* pathogenicity. Compared to blood-sourced *S. epidermidis*, traumatic endophthalmitis strains carried different virulence genes and causing intraocular infection which may be independent of biofilm formation. Overall, our study revealed the genetic diversity and pathogenicity of *S. epidermidis* and reported a comprehensive comparative analysis of different source genomes.

## Materials and Methods

### Strains

This study analyzed the whole genomes of 187 *S. epidermidis* isolates, including 11 ocular strain isolates from the Department of Laboratories, Eye Hospital of Wenzhou Medical University, Wenzhou, China, and 176 available genome sequences downloaded from the GenBank database (Sayers et al., 2020) of the National Center for Biotechnology Information (NCBI)^1^ and PATRIC v3.6.10,^2^ with detailed information showed in Supplementary Table 1. The 187 *S. epidermidis* strains were selected to represent known diverse niches and the host health states. We collected 17 ocular *S. epidermidis* strains, 46 blood-sourced strains, 22 respiratory strains, 45 skin-sourced strains, and 46 clinical strains with unknown sources of host niches defined as “group clinics,” of the remaining *S. epidermidis* strains belonged to the “Others” group. The “Respiratory” group consists of isolates from the nares, lungs, pharyngeal exudate, sputum, and bronchoalveolar lavage. Catheter and oral isolates and the reference genomes of strains ATCC14990 and RP62A were classified in the “Others” group. A total of 33 and 75 strains had a definite health or disease state, respectively.

### Whole-Genome Sequencing, Assembly, Gene Predictions, and Functional Annotations

**Figure 1:**
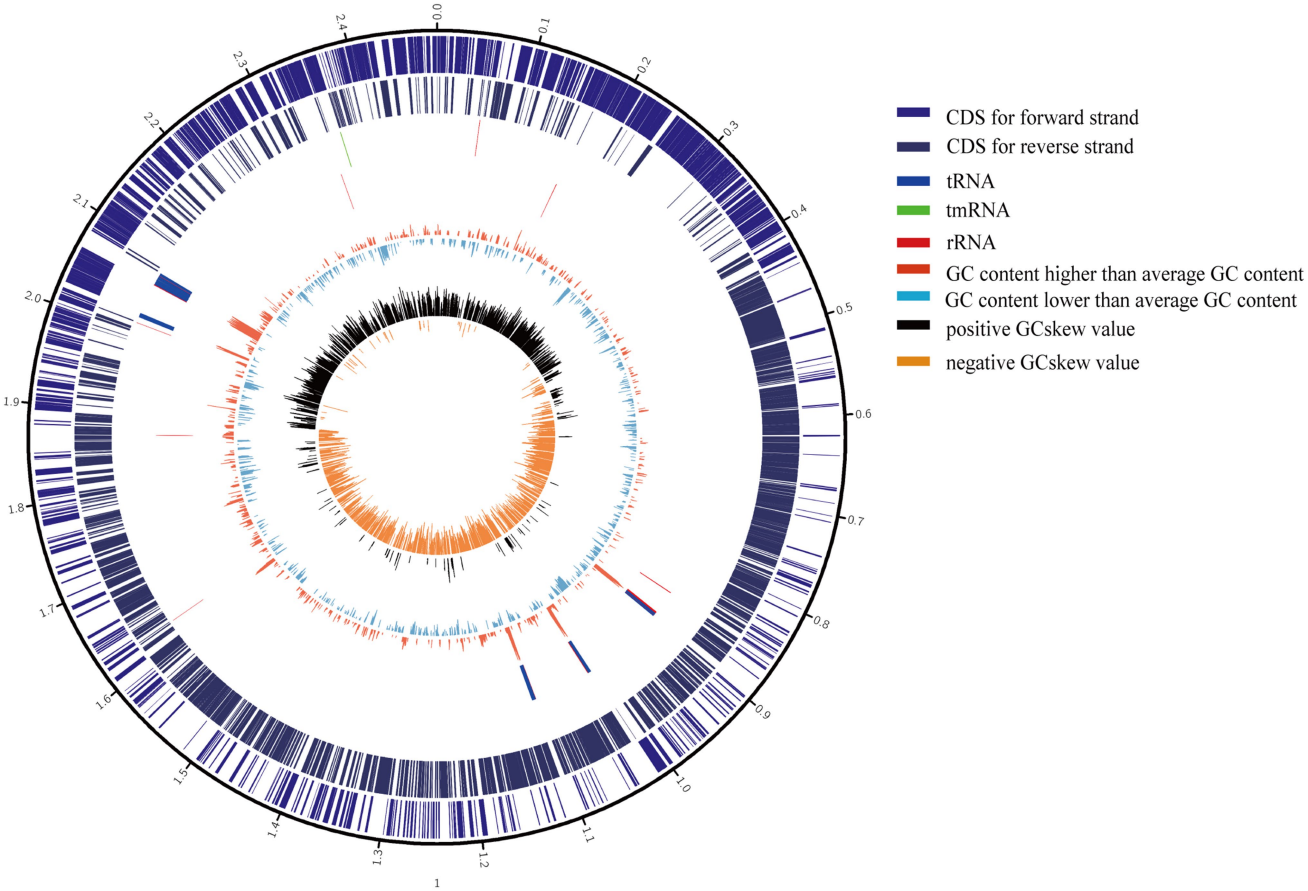
Circular genome maps of representative ocular strain. From the outer to the inner circle: (1) scale marks of genomes; (2) assigned clusters of orthologous group (COG) classes of protein-coding genes (CDSs) on the forward strand; (3) reverse strand CDSs; (4) tRNA (blue) and rRNA (red) genes on the forward strand; (5) tRNA (blue) and rRNA (red) genes on the reversed strand; (6) GC content (swell fire red/sky blue indicates higher/lower G+C compared with the average G+C content); (7) GC skew (black/orange indicate positive/negative values).

The 11 *S. epidermidis* isolates collected from ocular sources were cultured in 5ml of brain heart infusion broth (BHI) +5% fetal bovine serum (FBS, Gibco) with shaking at 250rpm and 37°C for 12–16h. DNA was extracted using a GENEray Bacterial Genome DNA Mini Kit (GENEray, Shanghai, China) following the manufacturer’s protocol. Whole-genome sequencing was performed by Berry Genomics Co., Ltd., Beijing, China, using PacBio SMRT Technology. SMRTbell libraries were prepared using the SMRTbell Express Template Prep Kit 2.0 (PacBio kit). Long read data were assembled by canu 2.1.1 (Koren et al., 2017), and assembly polisher was used with pbmm2 (v1.4.0) and gcpp (v2.0.0) *via* the bioconda package. Quast (v5.0.2; Gurevich et al., 2013), checkM (v1.1.3; Parks et al., 2015), and busco (v5.0.0; Manni et al., 2021) were used to assess the quality of assembled genomes. Coverage depth ultimately counted by pbmm2 mapping the draft genomes to raw subreads. The genes of all genomes, including the 11 ocular *S. epidermidis* genomes sequenced in this study and 176 obtained from NCBI, were predicted and annotated by Prokka (v1.13; Seemann, 2014). The sequences and annotation details of 11 ocular strains were listed in Supplementary Table 1, and a representative assembled genome were chosen to draw a genomics map with Circos (Krzywinski et al., 2009) to illustrate the genome characterization (Figure 1).

### Pan-Genome Analysis

Pan-genome analysis was carried out for all 187 genomes and the genomes of *S. epidermidis* isolate from different niches by BPGA v1.3 (Chaudhari et al., 2016) using default parameters, and 50% sequence identity as the cutoff for clustering identity was applied to USEARCH. The annotation files generated by Prokka were provided to BPGA as input. The clusters of orthologous groups (COGs) of the core sequence, accessory sequence, and unique sequence after Pan-genome analysis were further verified by performing functional annotations in the EggNOG Database (v5.0; Huerta-Cepas et al., 2019) *via* eggnog-mapper (v2.0; Huerta-Cepas et al., 2017) with the Diamond parameter.

### Characterization of *Staphylococcus epidermidis* Strains

kSNP3.1 (v3.1.2; Gardner et al., 2015) was used to build the phylogenetic tree, a validated method without alignment by 19 k-mer length, and a total of 8,760 core SNPs were identified. Then, a maximum likelihood core SNP tree was constructed by RAxML (v8.2.12; Stamatakis, 2014) with 100 bootstraps. The phylogenetic tree was visualized and beautified by online itol (v6.0; Letunic and Bork, 2021).^3^

Multilocus sequence typing (MLST) of 187 *S. epidermidis* strains was performed with the MLST 2.0 online server (Larsen et al., 2012).^4^ Online SCC*mec*Finder 1.2^5^ was used to identify SCC*mec* elements in sequenced *S. epidermidis* isolates.

ResFinder (v2.1; Zankari et al., 2012) and Resistance Gene Identifier (RGI; v5.1.1) of the comprehensive antibiotic resistance database (CARD; 2020; Alcock et al., 2020) were employed to identify antimicrobial resistance genes (AMRs). The resistance genes were verified by the paper diffusion method (MHA) and broth dilution method. The BLASTp program was used to search all protein sequences of 187 *S. epidermidis* strains against the Virulence Factor Database (VFDB; Feb, 2021; Liu et al., 2019).^6^ Compared with the virulence genes in the database at an *e*-value <1e-10, only query genes with an identity higher than 40% and a coverage higher than 70% were considered potential virulence genes (Nourdin-Galindo et al., 2017). Functional annotations of virulence factor were based on the categories and subcategories presented in VFDB. An alignment for each of the extracted candidate virulence genes was constructed using Clustal Omega (v1.2.4; Sievers et al., 2011) and visualized by ESPript (v3.0; Robert and Gouet, 2014).

### Statistical Analyses

The significance of core gene and accessory gene abundance in COG categories was examined using Fisher’s exact test. The disease-associated and ocular-associated predicted accessory genes with known function and annotated virulence genes were analyzed using Fisher’s exact tests and the FDR correction of values of *p*. All statistical analyses were carried out using the R package (version: 4.0.2). A value of *p*<0.05 was regarded as statistically significant.

## Results

### Pan-Genome and Functional Characterization of *Staphylococcus epidermidis* From Different Sources

**Figure 2:**
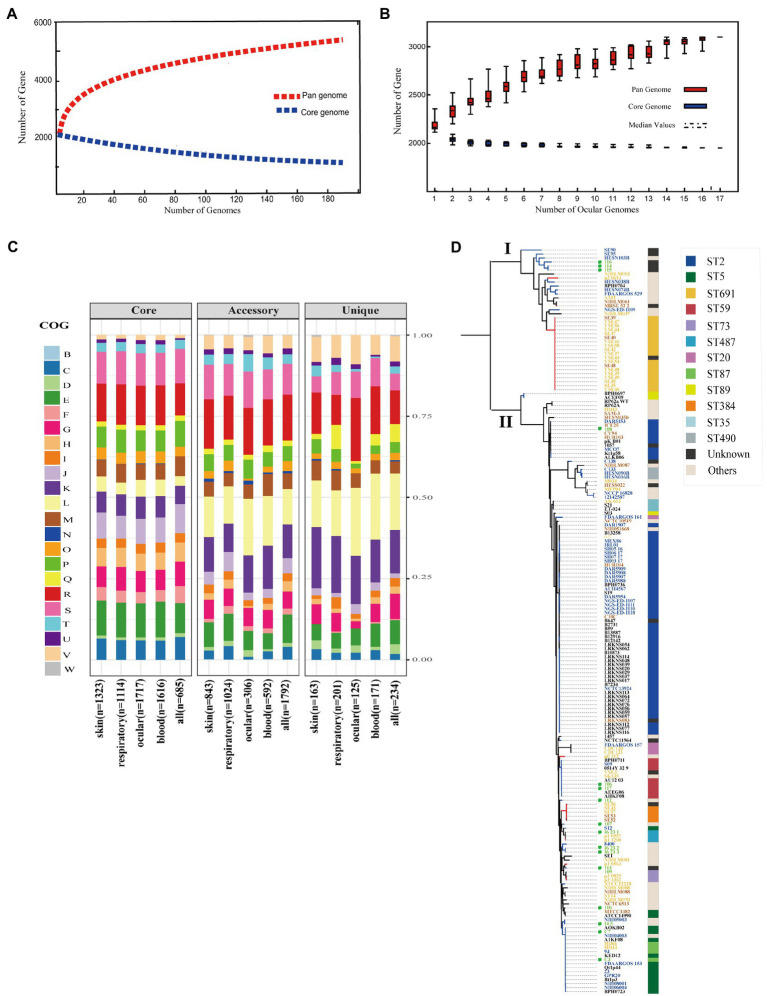
Pan-genome and phylogenetic feature of *Staphylococcus epidermidis* from different host niches and health state. **(A)** Gene accumulation curves for pan-genome (red) and core-genome (blue) of collected all strains. **(B)** Gene accumulation curves for pan-genome (red) and core-genome (blue) of ocular sources strains. **(C)** COG functional categories from the pan-genomes within strains from different niches. Involved COG categories are as follows: [B]Chromatin structure & dynamics; [C]Energy production & conversion; [D]Cell cycle control, cell division, chromosome partitioning; [E]Amino acid transport & metabolism; [F]Nucleotide transport & metabolism; [G]Carbohydrate transport & metabolism; [H]Coenzyme transport & metabolism; [I]Lipid transport & metabolism; [J]Translation, ribosomal structure & biogenesis; [K]Transcription; [L]Replication, recombination & repair; [M]Cell wall/membrane/envelope biogenesis; [N]Cell motility; [O]Post−translational modification, protein turnover & chaperones; [P]Inorganic ion transport & metabolism; [Q]Secondary metabolites biosynthesis, transport & catabolism and; [T]Signal transduction mechanisms; [U]Intracellular trafficking, secretion & vesicular transport; [V]Defense mechanisms; [W]Extracellular structures. Poorly characterized COG categories contains [R]General function prediction only and [S]Function unknown. **(D)** Phylogenetic core SNP maximum likelihood tree was constructed for 187 genomes. The blue branch represents strains from diseased hosts, while heathy source strains put color on red. Different color of strains ID stand for different host niches: blood (blue), ocular (green), skins (yellow), and respiratory tract (brick-red). Legends on the left stand for colors of sequence type (ST) from multilocus sequence typing. Ocular strains marked in green circle.

Although several studies reported pan-genomes characterization of *S. epidermidis* (Conlan et al., 2012), the core and pan-genome features of ocular strains are lacking. To identify the pan-genomic characteristics of *S. epidermidis* from ocular sources and discrepancies among different sources, we determined overall genetic similarities and differences among ocular *S. epidermidis* and all 187 strains. Gene accumulation curves (Tettelin et al., 2005; Figures 2A,B) showed that the number of core genomes fits an exponential decay curve that plateaus at 749 and 1,931 genes, respectively, while the pan-genome data fit a power law curve (*y*=*a·x^b^*), indicating an open pan-genome in which each genome sequence added several new genes, as reported (Conlan et al., 2012). To a certain degree, the capacity of acquiring exogenous DNA of the organism partially determines the pan-genome state (“open” or “close”; Diene et al., 2013), especially for species living within a bacterial community, such as skin inhabitants.

The function of the genes within the pan-genome of all strains from different sources was investigated by assigning all gene clusters to COGs (Galperin et al., 2021; Figure 2C). For the entirety, the results revealed a total of 685/749 core genes, 1,792/2,783 accessory genes, and 234/468 unique genes among all 187 *S. epidermidis* strains annotated to COG categories. Specifically, by Fisher’s exact test (FDR<0.05), 5 of 22 COG categories were significantly enriched in core genes and almost associated with metabolism and biogenesis: energy production and conversion, nucleotide transport and metabolism, coenzyme transport and metabolism, translation, ribosomal structure and biogenesis, and inorganic ion transport and metabolism. Additionally, while replication, recombination, and repair were enriched in both accessory and unique genes, secondary metabolite biosynthesis, transport, and catabolism and defense mechanisms were enriched in unique genes. Second, we compared the enrichment of COG function among strains from different sources, including the blood, eyes, respiratory tract, and skin, and showed similar results: core genes related to metabolism and biogenesis. However, genes associated with defense mechanisms were enriched in accessory genes of all strains except eye-sourced ones. In addition, transcription was significantly enriched in accessory genes of blood-sourced strains, while other sources were enriched in unique genes. In summary, compared to the core genes enriched in biogenesis and metabolism, the enrichment of replication, recombination and repair, defense mechanisms, and transcription among accessory genes and unique genes were driven by diversity in recombinase and integrase, ABC-type multidrug transporters, and transcriptional regulators, respectively. These abundant genes often appear to transfer horizontally between strains, leading to the spread of virulence and resistance genes between strains, which affects the pathogenicity of bacteria (Jackson et al., 2011).

### Phylogenetic Relationship and Associated Typing Among *Staphylococcus epidermidis*

To infer the phylogenetic relationship of the *S. epidermidis* strains from different sources, we used 8,760 core SNPs to build a single-nucleotide polymorphism-based phylogenetic tree. As expected, the 187 *S. epidermidis* isolates formed two distinct groups termed I and II, as previously reported (Conlan et al., 2012; Zhou et al., 2020; Figure 2D), suggesting the presence of two founder lineages. Moreover, we found that ST2 and ST5 strains were present in Cluster II and correlated with definite disease hosts or clinical strains, while ST691 strains were present in Cluster I associated with healthy skin with the same genetic distance.

It is worth mentioning that although the ocular isolates varied in phylogeny, they all had a close distance to different sources from diseased hosts in both clades, which confirmed that the ocular strains that we collected were pathogenic. However, the ST typing of ocular strains was highly diverse and included rare specimens: 4/11 strains that we collected were unknown.

*Staphylococcus epidermidis* contains SCC*mec*, called methicillin-resistant *S. epidermidis* (MRSE; Conlan et al., 2012). SCC*mec* is a mobile genetic element defined by combinations of *mec* gene complexes, cassette recombinases, and accessory genes and carries the key determinant for broad-spectrum beta-lactam resistant *mecA* gene of *Staphylococcus* species (Conlan et al., 2012; McManus et al., 2015). We found that the SCC*mec* element (SCC*mec* type) was present in 94/187 strains. Interestingly, almost all SCC*mec*-positive strains (96.8%, 91/94) fell in Cluster II, indicating that *S. epidermidis* carrying the SCC*mec* element may be more pathogenic relative to SCC*mec*-negative strains.

### The Virulence Characteristics of *Staphylococcus epidermidis*

**Figure 3:**
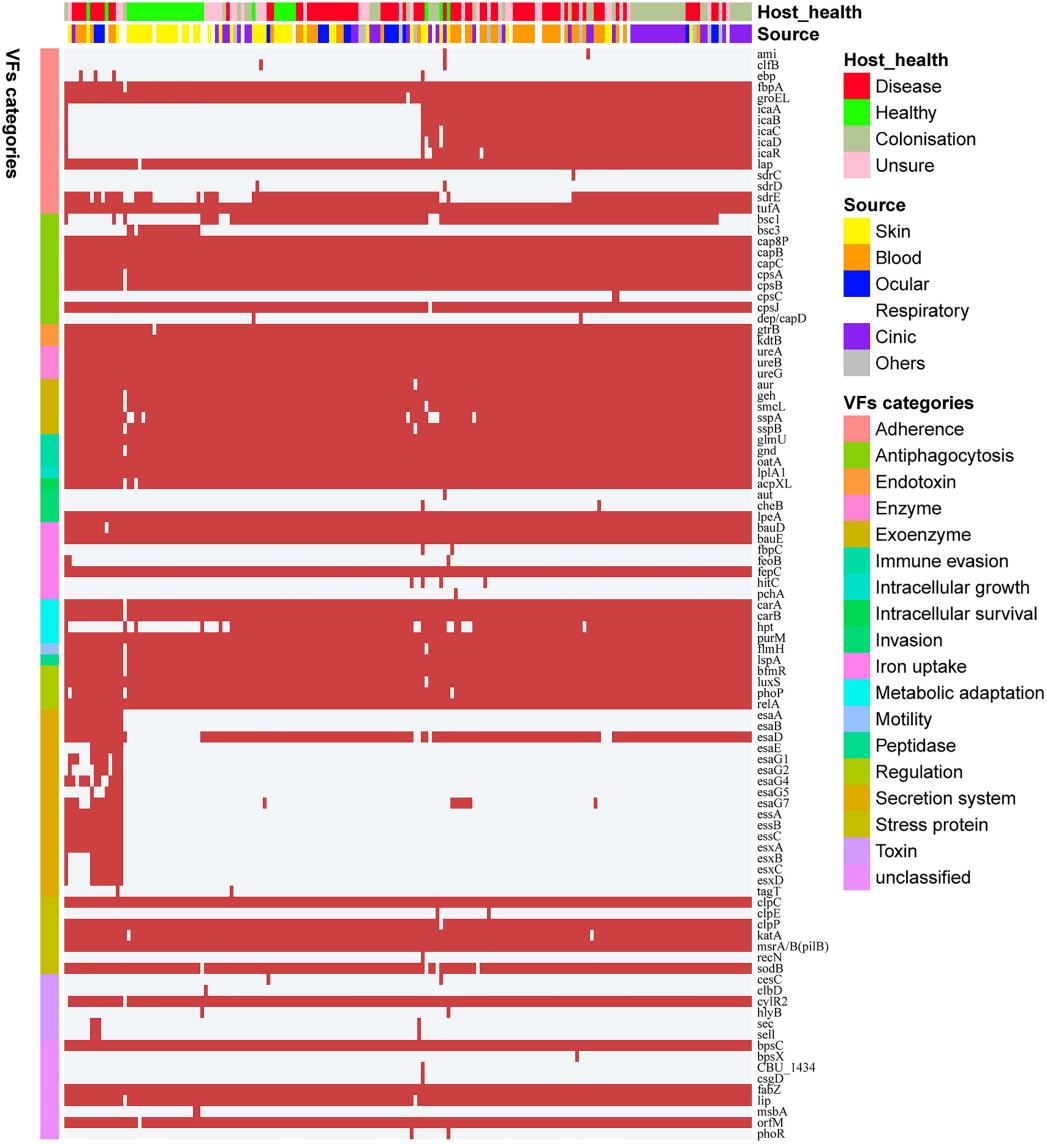
Heatmap of virulence genes in all 187 *S. epidermidis* strains. The red square indicates the presence of the gene, while the white square indicates absence. Legends on the right stand for colors of different host healthy state, isolates source, and virulence factor categories.

*Staphylococcus epidermidis* is an opportunistic pathogen that harbors many virulence genes. To analyze the difference in pathogenic potentials among *S. epidermidis* isolates from different sources, 99 genetic loci related to virulence were identified based on the VFDB. These loci were grouped into 18 categories (Figure 3), including 21 (21/99, 21.2%) virulence genes that coexisted in all *S. epidermidis* genomes. Surprisingly, we identified several virulence genes, including *icaABCDR*, *hpt*, and *esaD*, that were strongly associated with *S. epidermidis* isolates from disease sources (Fisher’s exact test, FDR<0.01). Polysaccharide intercellular adhesion (*icaABCD*) genes that encode biofilm-associated genes for poly-*N*-acetylglucosamine synthesis were found in 50.7% (38/75) of isolates from disease sources. Notably, only 2 of 33 isolates from healthy tissues contained the *icaABCD* gene, an observation different from previous studies showing that this gene was present in 60% of commensal isolates. Pairwise comparison of the enrichment of virulence genes between ocular-sourced strains and strains from other niches showed 30 *icaABCD* genes in all 46 blood-sourced strains and 3 out of 17 ocular-sourced strains. We also found that the *sdrE* gene encoding the Ser-Asp-rich protein was enriched in ocular-sourced strains (16/17) compared to blood-sourced strains (24/46; Fisher’s exact test, FDR<0.05). Two toxin genes, *sell* and *sec*, which were present only in two *S. epidermidis* strains isolated from blood (SE90 and SE95), which reported previously (Sun et al., 2020), were also identified in two ocular strains. Together with the results from phylogenetic analysis showing that these strains had a close evolutionary distance, implying that they may have the similar founder lineages.

### Gene Differences Between Genomes of Isolates From Healthy and From Disease Sources

**Figure 4:**
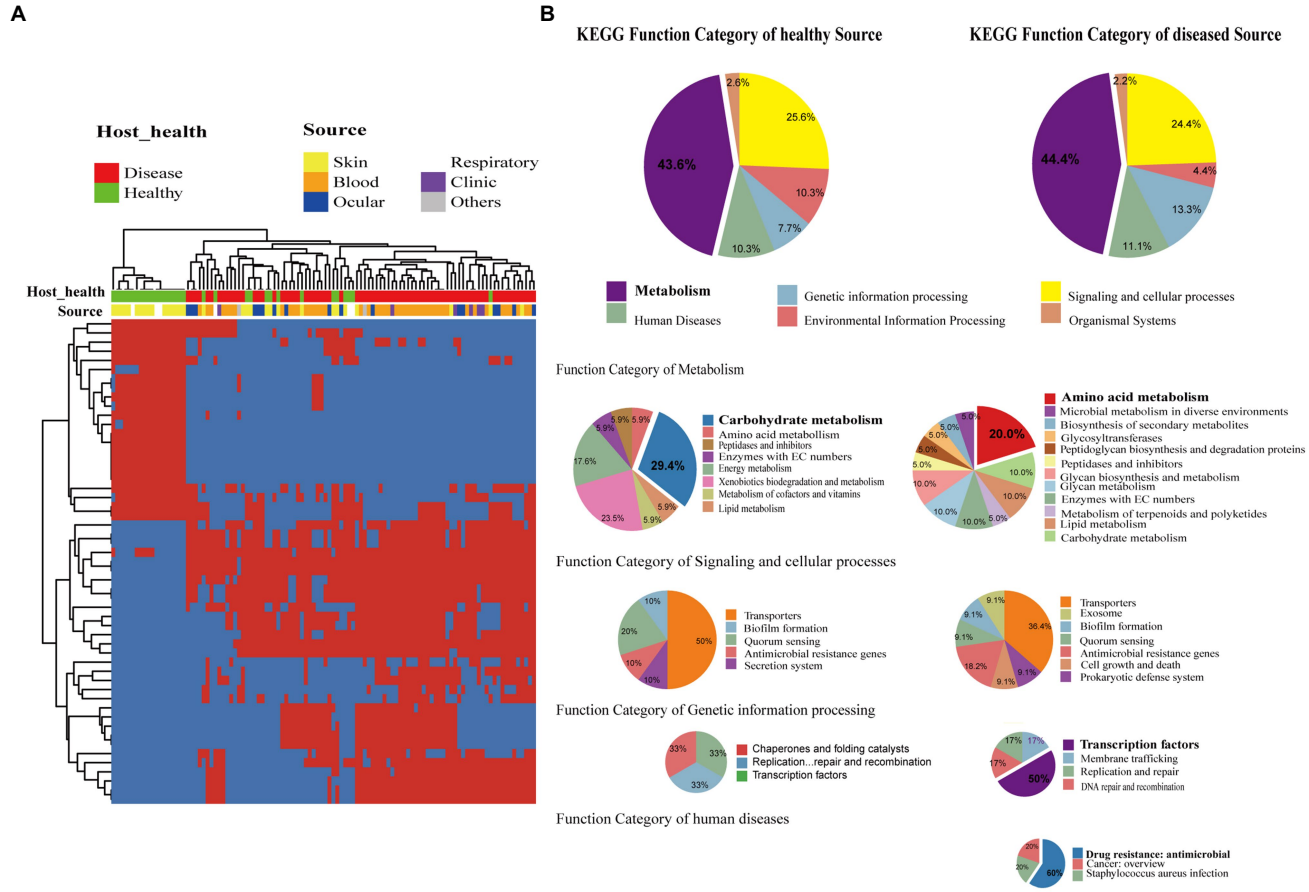
Gene function difference between healthy and disease source genomes. **(A)** Heatmap of enriched genes (Fisher’s exact test, adjust *p*<0.05) between disease and healthy sources strains. Only the accessory genes were shown. Gene clusters present in all genomes (core gene) or present in only a single genome (unique genes) are omitted. The red color stands for genes that existed and the blue color for missing ones. **(B)** Pie graph of KEGG function related with enriched genes from different health sources (Left are healthy source and Right are disease source). Bigger pie represented KEGG function proportion of the enriched genes showed in **(A)**, while three smaller pie were account for function categories of metabolism, genetic information process, signaling and cellular processes, human diseases, respectively.

*Staphylococcus epidermidis* is a coagulase-negative and Gram-positive *staphylococcus* found in skin and mucosa microflora (Oh et al., 2016). It is also relatively highly abundant with high positive rates on the ocular surface and the second leading cause of nosocomial infections (Ziebuhr et al., 2006). We further investigated whether the presence of specific genes was significantly correlated with different sources of strains including isolates from different healthy hosts. We devoted to accessory genes defined as gene clusters neither present in all genomes nor exit in only a single genome. Distinctly, the genes were divided into two clusters, which strictly differentiated the disease group and healthy isolates (Fisher’s exact test, FDR<0.01; Figure 4A). The two groups of genes were then functionally classified by KEGG function (Figure 4B). For both groups, metabolism genes accounted for the largest proportion, followed by signaling and cellular processes. Comparing the two groups in the subcategory of KEGG function, disease-associated genes had a larger proportion involved in amino acid metabolism, antimicrobial resistance, and transcription factors, while carbohydrate metabolism had a high ratio in healthy-related genes.

**Figure 5:**
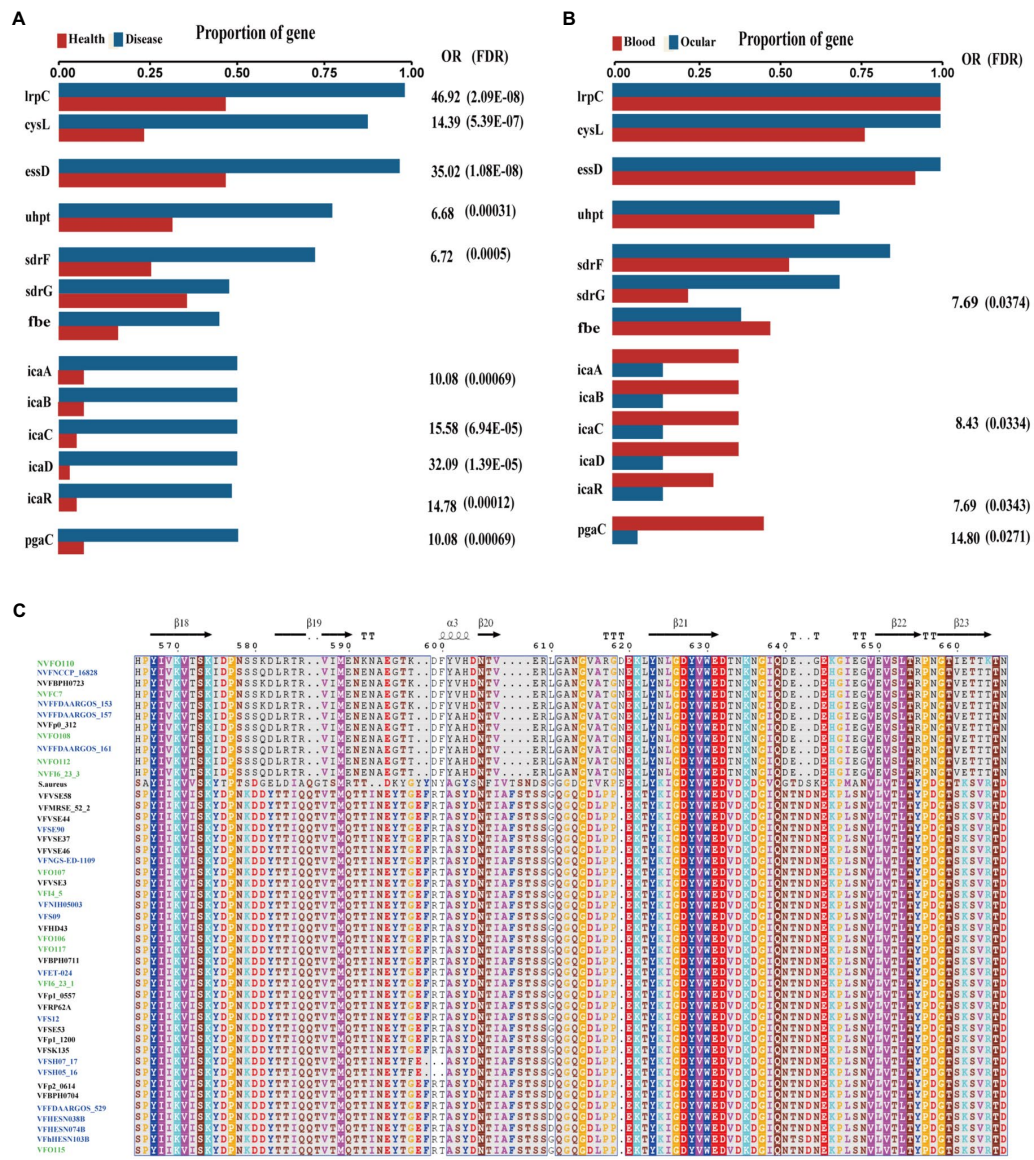
Key genes associated with diseased source pathogens. **(A)** Frequency of key genes among bacteria isolated from different health state. OR, odds ratio for association between the presence of the genes and disease vs. health source; values of *p* were calculated using Fisher’s exact test. **(B)** Frequency of key genes among bacteria isolated from different human source. OR, odds ratio for association between the presence of the genes and disease vs. health source; values of *p* were calculated using Fisher’s exact test. **(C)** Multiple alignment of virulence gene sdrG and non-virulence gene sdrG protein sequences across the collected blood source *S. epidermidis* (blue), intraocular isolates (green), *Staphylococcus aureus* and sdrG positive skin source strains.

Among the differentially expressed genes between strains from diseased and healthy sources, we found some key disease-related genes, including the virulence genes *essD*, *uhpt*, *sdrF*, *sdrG*, *fbe*, and *icaABCDR* and the transcriptional regulators *lrpC* and *cysL* (Figure 5A). *SdrF*, *sdrG,* and *fbe,* which are microbial surface components recognizing adhesive matrix molecules (MSCRAMMs), showed homology with the virulence gene *sdrE*. Still, not all of the gene sequences were identified as virulence factors. By multiple alignment with Clustal omega, we found that compared to both the virulence-associated *sdrG* of *S. epidermidis* from and *sdrG* of *Staphylococcus aureus*, the non-virulence-associated *sdrG* of *S. epidermidis* had mutation in the region encoding a β-strand structure (Figure 5C), which may have reduced virulence.

Interestingly, comparing the genes between the isolates from ocular sites and other sites, we found that biofilm-related genes *icaABCDR* and *pgaC* were markedly enriched in isolates from blood but not in isolates from ocular tissue (Figure 5B). Moreover, although *sdrG* seemingly was enriched in isolated from ocular sites, the mutation rate within the β-strand reached 42.86% (6/14), which may result in a reduced adherence of the microbes to the extracellular matrix of the host, further suggesting that biofilm formation may not be a direct factor for intraocular infection of *S. epidermidis*.

### Antimicrobial Resistance Across *Staphylococcus epidermidis*

**Figure 6:**
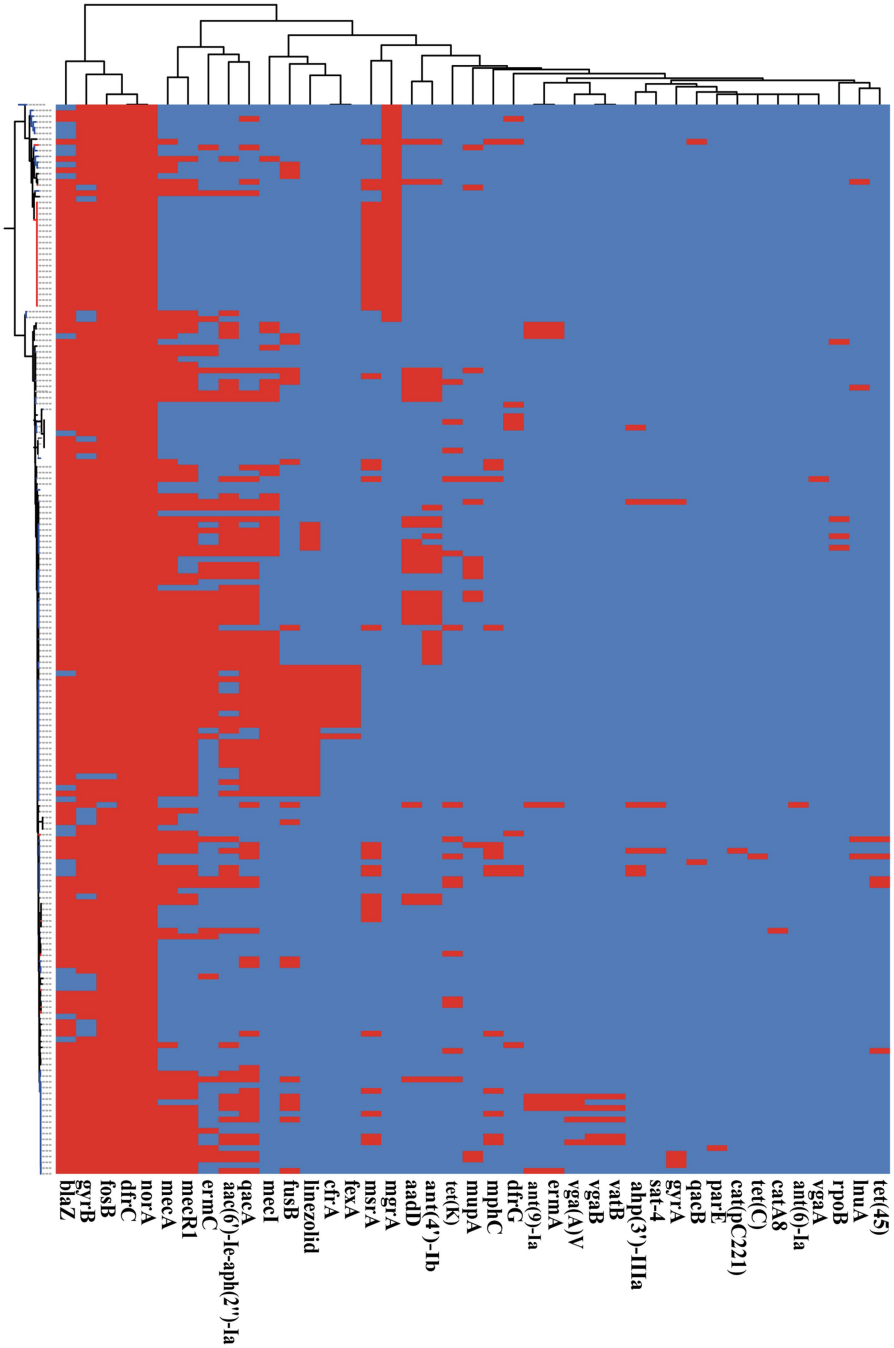
The presence of antibiotic resistant genes across *S. epidermidis* strains. Heatmap indicates of 41 antibiotic resistant genes involved. The red color stands for genes that existed and the dark blue color for missing ones.

Antimicrobial resistance is very common among *S. epidermidis* isolates and contributes to the persistence of clinical infection and often limits treatment options (Kleinschmidt et al., 2015). To investigate antimicrobial resistance across *S. epidermidis*, we analyzed all known AMR genes within our 187 genomic data sets. Our analysis of ResFinder and CARD databases found 41 different genes involved in resistance to 19 antibiotics (Figure 6). Nearly, all isolates carried at least three antibiotic resistance genes. Among the genes involved in AMR, our data showed that two genes were conserved in all strains, *norA*, which is associated with fluoroquinolone antibiotics that belongs to the AMR gene family with major facilitator superfamily (MFS) antibiotic efflux pumps, and *dfrC*, a diaminopyrimidine antibiotic-associated gene. This result was consistent with the data in the CARD.^7^^,^^8^

Based on Fisher’s exact test of strains from different host health states and niches, we found that the methicillin resistance genes *mecA* and *mecR1* (FDR<0.001), fluoroquinolone antibiotic gene *qacA* (FDR<0.005), and aminoglycoside antibiotic gene *aac(6′)-Ie-aph(2″)-Ia* (FDR<0.001) were enriched in strains isolated from disease hosts. The presence of *mecA* was consistent with the SCC*mec*-positive strains because the mobile genetic element SCC*mec* carried *mecA*. Antibiotic susceptibility testing using oxacillin and cefoxitin to assess resistance to methicillin. The results showed that the intraocular strain oxacillin resistance rate was 5/11 (Table 1), which was consistent with the rate of the results for cefoxitin and *mecA* gene carriers. Moreover, strains from healthy skin had significant enrichment of the *msrA* and *mgrA* genes involved in multidrug resistance (FDR<0.001), while isolates from the ocular, blood, and respiratory tracts had no significantly enriched antibiotic resistance genes, suggesting that regardless of the isolation niches, none of these isolates were resistant to specific antibiotics at the genetic level.

**Table 1:** *Mec* gene carried rate and antibiotic susceptibility testing results of 11 ocular.

	O106	O107	O108	O109	O110	O112	O113	O114	O115	O116	O117
*mecA*	+	−	+	−	+	+	−	−	−	−	+
*mecR1*	+	−	−	−	−	−	−	−	−	−	+
Oxacillin	R	S	R	S	R	R	S	S	S	S	R
Cefoxitin	R	S	R	S	R	R	S	S	S	S	R

## Discussion

Coagulase-negative *Staphylococcus* is the most common pathogen in traumatic endophthalmitis (Durand, 2013). *Staphylococcus epidermidis*, the most common coagulase-negative Gram-positive *Staphylococcus*, colonizes the normal mucosa, skin flora, and intraocular tissue of humans and other mammals and is one of the major leading causes of clinical infections (Oh et al., 2016). What still needs to be discussed is whether *S. epidermidis* isolated from the tissues is a pathogen or a contaminant. Although the genome characteristics of *S. epidermidis* have been studied in recent years, a comprehensive understanding of the genomes of ocular-sourced strains is lacking. To the best of our knowledge, this is the first and largest collection of bacterial genome sequences isolated from patients with *S. epidermidis* intraocularly. By collecting, sequencing, and analyzing the genomes of 11 intraocular isolates and incorporating publicly available genomes of *S. epidermidis*, we gained insight into the phylogenetic and molecular characteristics of intraocular and other niche pathogens. The genetic differences between pathogenic and commensal *S. epidermidis* were also investigated comprehensively.

In this study, we compared the phylogenetic diversity and genome characteristics of *S. epidermidis* from different niches and different host health states. The host niches, including the eye, blood, skin, and respiratory tract, were from individuals with different healthy state. The 11 ocular-sourced *S. epidermidis* genomes sequenced revealed similar high-quality benchmark data, including genome size, GC content, and the number of predicted genes, similar to the data collected from public databases with a relatively compact genome with an average size of approximately 2.58Mb. Furthermore, the completeness of each whole-genome sequencing exceeded 99%, and the contamination level of the sequencing library was also very low. The high coverage of genome assembly allowed us to obtain a complete and accurate genome sequence. Consistent with the results of previous studies (Conlan et al., 2012; Sharma et al., 2018; Zhou et al., 2020), all 187 genomes, whether in the pan-genome analysis of all strains from various niches, including ocular sources, showed that the size of the *S. epidermidis* genome was relatively constant, extracted from a larger gene pool, indicating an increase in the “open” pan-genome, and some new genes were added in each genome sequence. To some extent, the pan-genomic state (“open” or “closed”) of an organism depends in part on its ability to obtain exogenous DNA (Diene et al., 2013). For example, the large number of genes involved in the mobile group results in frequent horizontal gene transfer (HGT) between staphylococcal stains, and the presence of mobile genetic elements such as SCC*mec*, ACME, and plasmids leads to an increase in the “open” pan-genome (Georgiades and Raoult, 2010). The phylogenetic tree constructed based on genome-wide core SNPs reveals important details that were not able to observe with traditional single-gene markers (16S rDNA) or MLST (Su et al., 2020). From the phylogenetic tree analysis, we found that 187 strains of *S. epidermidis* formed two distinct clusters with different pathogenic capacities. The clinically pathogenic strains were generally ST5 and ST2. In contrast, the ST691 strains were derived from healthy skin. The previously reported evolutionary distance between ST2 *S. epidermidis* was extremely short (Su et al., 2020) and was also observed in the ST5 and ST691 strains.

It is well established that *S. epidermidis* is a common human skin commensal. It is considered an opportunistic pathogen and causes infection when it breaks through the surface of the skin and enters the blood (Otto, 2009). *Staphylococcus epidermidis* is also an vital commensal on the ocular surface (Zhang et al., 2013), but it can enter the eyes to cause intraocular infection when injury occurs usually caused by trauma. By whole-genome analysis in this study, we have provided a powerful framework for redefining species clustering in the genus, locating genetic traits, and rating the importance of disease-causing genes based on their presence or absence in the genomes. By analyzing the genomes of *S. epidermidis* from different sources using comparative genomics methods, we identified the putative pathogenic marker genes *lrpC, cysL, essD, uhpt, sdrF, sdrG, fbe, and icaABCDR*. *lrpC* and *cysL* both encode helix-turn-helix (HTH)-type transcriptional regulators. The *lrp*-like regulatory factor consisted of a helix-turn-helix (HTH)-type *n*-terminal DNA-binding domain, which is connected to the C-terminal RAM domain (amino acid metabolism regulation) responsible for cofactor binding and oligomerization (Ettema et al., 2002). Thus, *lrpC* is a transcriptional regulator with a possible role in the regulating amino acid metabolism and the growth phase transition. *CysL* belongs to the LysR family transcriptional regulator. The LysR is a DNA-binding protein with a winged helix-turn-helix (wHTH) domain consisting of approximately 60 residues in the LysR-type transcription regulator (LTTR). LTTR is one of the most common regulatory factor families in prokaryotes. The c-terminus of the LysR protein often contains a regulatory domain with two subdomains, which participate in (1) coinducer recognition/reaction and (2) DNA binding and response (Henikoff et al., 1988). LTTRs can activate the transcription of operons and regulons involved in the regulation of various functions, such as amino acid biosynthesis, CO_2_ fixation, antibiotic resistance, virulence factor regulation, nitrogen-fixing bacterial nodulation, oxidative stress response, or aromatic compound catabolism. However, the specific functions of the transcriptional regulators CysL and LrpC in *S. epidermidis* remain to be determined.

In this work, the pathogenic marker genes *essD*, *uhpt*, *sdrF*, *sdrG*, *fbe*, and *icaABCDR* were identified as potential virulence genes. Interestingly, we found that the potential virulence genes *essD* and *uhpt* had high homology with *esaD* and *hpt* in *S. aureus*. *EsaD* is a nuclease toxin secreted by type VII secretion system, which may play a key role in bacterial competition (Cao et al., 2016). Hexose phosphate is an important carbon source in the cytoplasm of host cells (Park et al., 2015a). Bacterial pathogens invade, survive, and replicate in different host epithelial cells, utilizing hexose phosphate in the host cytoplasm to obtain energy and synthesize cell components through the hexose phosphate transport (HPT) system (Park et al., 2015a). The HPT system of *S. aureus*, which includes the *hptRS* (a new type of two-component regulatory system), *hptA* (a phosphate sensor), and *uhpT* (a hexose phosphate transporter) genes, allowing it survive in the host cell and may be an important target for the development of new anti-*staphylococcal* therapies (Park et al., 2015a). The potential virulence genes essD and uhpt of *S. epidermidis* identified in this study could be due to HGT between *Staphylococcus* strains; for instance, the genomes exchange between *S. epidermidis* and *S. aureus* during evolution. In fact, it was reported that multiple accessory genes of *S. epidermidis* were significantly associated with features of the contextual microbiome and could be generalized to new hosts (Zhou et al., 2020). Recently, Du et al. (2021) reported that some *S. epidermidis* may have gained the capacity to exchange DNA, such as an accessory tarIJLM gene cluster, *via S. aureus* phage.

*SdrF, sdrG, fbe*, and polysaccharide intercellular adhesion gene (*icaABCD*) are related to biofilm formation (Foster et al., 2014). Biofilm formation is the main virulence mechanism of *S. epidermidis* contributed to the persistence of clinical infections (Schoenfelder et al., 2010). Here, all seven genes encode adhesive molecules, which are well-known factors involved in biofilm formation. *SdrF, sdrG,* and *fbe* are a subset of MSCRAMMs (Foster et al., 2014). They are covalently anchored to the cell wall and characterized by a segment composed of repeated serine aspartate (SD) dipeptides (Vazquez et al., 2011). MSCRAMMs are bacterial surface proteins that mediate the adhesion of microorganisms to the host’s extracellular matrix components (Vazquez et al., 2011). Our sequence analysis showed that not all of the *sdr* gene sequences were identified as virulence factors. The sequence of *sdrF* in strains associated with virulence was very different from that of non-virulence strains, including the difference in sequence length, while only the β chain mutation occurred between the two *sdrG* genes. We speculated that the occurrence of these mutations may reduce the virulence of *sdrG*, and it was clear that mutagenesis is overrepresented in intraocular isolates compared to blood isolates. Our enrichment analysis indicated that it was possible to differentiate the intralocular pathogenic strains from those from blood, with biomarkers related to biofilm formation. For example, polysaccharide intercellular adhesion *icaABCD* was enriched in blood but not in ocular tissue. This may be due to the fact that *S. epidermidis* is more likely to form biofilms on medical devices such as catheters and artificial heart valves (Wisplinghoff et al., 2004). Bacterial cells on these devices can break away from the biofilm and enter the bloodstream, leading to bacteremia, increasing morbidity, and potential mortality (Wisplinghoff et al., 2004). On the other hand, this may also suggest that the intraocular infection of *S. epidermidis* is not directly related to its ability to form biofilm. A larger data set and further experiments are required to test this hypothesis.

*Staphylococcus epidermidis* has been found to be a treasure trove of antibiotic resistance (Conlan et al., 2012). Through a rare HGT event, its determinants of toxicity were shared with other more pathogenic species, such as *S. aureus*, as reported in previous studies (Qin et al., 2016). In our study, we found that 94/187 (50.2%) of the collected *S. epidermidis* species were MRSE with the *mecA* gene and SCC*mec* elements. In particular, the SCC*mec* cassette conferring β-lactam resistance was often transferred between staphylococcal strains, enabling them to rapidly evolve and adapt to antibiotic selection pressures to gain additional competitive advantages. This provided strong support to the notion that pathogens influence the risk of infection by the background microbiota through HGT of the pathogenic host, thereby increasing the risk of infection in other parts of the body, such as methicillin-resistant *S. aureus* in the nasal cavity affecting *S. epidermidis*-infected endophthalmitis (Su et al., 2020).

## Conclusion

Our study provided information on the molecular characteristics of different pathogenic *S. epidermidis* isolated from different host niches around the world, including the ocular strains that had been overlooked previously. Pan-genome and phylogenetic analyses demonstrated that *S. epidermidis* had an open pan-genome, and the two founder lineages had different pathogenicity. Interestingly, MRSE strains were concentrated in the pathogenic clade. Although the endophthalmitis-associated *S. epidermidis* isolated in this study was relatively dispersed in evolution, they are closer to the clinical pathogenic strains, which demonstrate the nature of their pathogenicity. Based on comparative genomics, we identified eight potential biomarkers related to strain pathogenicity and provided evidence that HGT may occur between *Staphylococcus* strains. Moreover, we reported the complete genome sequence of *S. epidermidis* that caused traumatic endophthalmitis and found that those strains causing intraocular infection may be independent of biofilm formation. Overall, this study revealed genetic diversity and pathogenic differences in different sources of *S. epidermidis*.

## Data Availability Statement

The sample and sequence data obtained in this study have been submitted to the NCBI BioSample and Sequence Read Archive (SRA) under BioProject accession number PRJNA753005.

## Author Contributions

SL, BS, JS, YL, and MZ contributed to conception and design of the study. BS, XM, TW, and JX collected the strains and extracted DNA. SL and XG collected databases and performed the bioinformatics analysis. YX and YG performed the drug sensitive test. SL, XS, and MZ wrote the manuscript. All authors contributed to the article and approved the submitted version.

## Conflict of Interest

The authors declare that the research was conducted in the absence of any commercial or financial relationships that could be construed as a potential conflict of interest.

## Publisher’s Note

All claims expressed in this article are solely those of the authors and do not necessarily represent those of their affiliated organizations, or those of the publisher, the editors and the reviewers. Any product that may be evaluated in this article, or claim that may be made by its manufacturer, is not guaranteed or endorsed by the publisher.

## Footnotes

^1^ http://www.ncbi.nlm.nih.gov

^2^ https://www.patricbrc.org

^3^ https://itol.embl.de/

^4^ https://cge.cbs.dtu.dk/services/MLST/

^5^ https://cge.cbs.dtu.dk/services/SCC*mec*Finder/

^6^ http://www.mgc.ac.cn/VFs/

^7^norA: https://card.mcmaster.ca/ontology/36530

^8^dfrC: https://card.mcmaster.ca/ontology/39299

## References

[ref1] Alcock B. P. Raphenya A. R. Lau T. T. Y. Tsang K. K. Bouchard M. Edalatmand A.. (2020). CARD 2020: antibiotic resistome surveillance with the comprehensive antibiotic resistance database. Nucleic Acids Res. 48, D517–D525. doi: 10.1093/nar/gkz935, PMID: 31665441 PMC7145624

[ref2] Alekshun M. N. Levy S. B. (2007). Molecular mechanisms of antibacterial multidrug resistance. Cell 128, 1037–1050. doi: 10.1016/j.cell.2007.03.004, PMID: 17382878

[ref3] Al-Omran A. M. Abboud E. B. Abu El-Asrar A. M. (2007). Microbiologic spectrum and visual outcome of posttraumatic endophthalmitis. Retina 27, 236–242. doi: 10.1097/01.iae.0000225072.68265.ee, PMID: 17290207

[ref4] Asbell P. A. Sahm D. F. Shaw M. Draghi D. C. Brown N. P. (2008). Increasing prevalence of methicillin resistance in serious ocular infections caused by *Staphylococcus aureus* in the United States: 2000 to 2005. J. Cataract Refract. Surg. 34, 814–818. doi: 10.1016/j.jcrs.2008.01.016, PMID: 18471638

[ref5] Becker K. Heilmann C. Peters G. (2014). Coagulase-negative staphylococci. Clin. Microbiol. Rev. 27, 870–926. doi: 10.1128/CMR.00109-13, PMID: 25278577 PMC4187637

[ref6] Bhagat N. Nagori S. Zarbin M. (2011). Post-traumatic infectious endophthalmitis. Surv. Ophthalmol. 56, 214–251. doi: 10.1016/j.survophthal.2010.09.002, PMID: 21397289

[ref7] Bispo P. J. Hofling-Lima A. L. Pignatari A. C. (2014). Characterization of ocular methicillin-resistant *Staphylococcus epidermidis* isolates belonging predominantly to clonal complex 2 subcluster II. J. Clin. Microbiol. 52, 1412–1417. doi: 10.1128/JCM.03098-13, PMID: 24523473 PMC3993641

[ref8] Cao Z. Casabona M. G. Kneuper H. Chalmers J. D. Palmer T. (2016). The type VII secretion system of *Staphylococcus aureus* secretes a nuclease toxin that targets competitor bacteria. Nat. Microbiol. 2:16183. doi: 10.1038/nmicrobiol.2016.183, PMID: 27723728 PMC5325307

[ref9] Chaudhari N. M. Gupta V. K. Dutta C. (2016). BPGA- an ultra-fast pan-genome analysis pipeline. Sci. Rep. 6:24373. doi: 10.1038/srep24373, PMID: 27071527 PMC4829868

[ref10] Cogen A. L. Yamasaki K. Muto J. Sanchez K. M. Crotty Alexander L. Tanios J.. (2010a). *Staphylococcus epidermidis* antimicrobial delta-toxin (phenol-soluble modulin-gamma) cooperates with host antimicrobial peptides to kill group A *Streptococcus*. PLoS One 5:e8557. doi: 10.1371/journal.pone.0008557, PMID: 20052280 PMC2796718

[ref11] Cogen A. L. Yamasaki K. Sanchez K. M. Dorschner R. A. Lai Y. Macleod D. T.. (2010b). Selective antimicrobial action is provided by phenol-soluble modulins derived from *Staphylococcus epidermidis*, a normal resident of the skin. J. Invest. Dermatol. 130, 192–200. doi: 10.1038/jid.2009.243, PMID: 19710683 PMC2796468

[ref12] Conlan S. Mijares L. A. Program N. C. S. Becker J. Blakesley R. W. Bouffard G. G.. (2012). *Staphylococcus epidermidis* pan-genome sequence analysis reveals diversity of skin commensal and hospital infection-associated isolates. Genome Biol. 13:R64. doi: 10.1186/gb-2012-13-7-r64, PMID: 22830599 PMC4053731

[ref13] Cornut P. L. Youssef E. B. Bron A. Thuret G. Gain P. Burillon C.. (2013). A multicentre prospective study of post-traumatic endophthalmitis. Acta Ophthalmol. 91, 475–482. doi: 10.1111/j.1755-3768.2011.02349.x, PMID: 22313810

[ref14] Diene S. M. Merhej V. Henry M. El Filali A. Roux V. Robert C.. (2013). The rhizome of the multidrug-resistant Enterobacter aerogenes genome reveals how new “killer bugs” are created because of a sympatric lifestyle. Mol. Biol. Evol. 30, 369–383. doi: 10.1093/molbev/mss236, PMID: 23071100

[ref15] Du X. Larsen J. Li M. Walter A. Slavetinsky C. Both A.. (2021). *Staphylococcus epidermidis* clones express *Staphylococcus aureus*-type wall teichoic acid to shift from a commensal to pathogen lifestyle. Nat. Microbiol. 6, 757–768. doi: 10.1038/s41564-021-00913-z, PMID: 34031577

[ref16] Durand M. L. (2013). Endophthalmitis. Clin. Microbiol. Infect. 19, 227–234. doi: 10.1111/1469-0691.12118, PMID: 23438028 PMC3638360

[ref17] Egert M. Simmering R. Riedel C. U. (2017). The association of the skin microbiota with health, immunity, and disease. Clin. Pharmacol. Ther. 102, 62–69. doi: 10.1002/cpt.698, PMID: 28380682

[ref18] Ettema T. J. Brinkman A. B. Tani T. H. Rafferty J. B. Van Der Oost J. (2002). A novel ligand-binding domain involved in regulation of amino acid metabolism in prokaryotes. J. Biol. Chem. 277, 37464–37468. doi: 10.1074/jbc.M206063200, PMID: 12138170

[ref19] Foster T. J. (2017). Antibiotic resistance in *Staphylococcus aureus*. Current status and future prospects. FEMS Microbiol. Rev. 41, 430–449. doi: 10.1093/femsre/fux007, PMID: 28419231

[ref20] Foster T. J. Geoghegan J. A. Ganesh V. K. Hook M. (2014). Adhesion, invasion and evasion: the many functions of the surface proteins of *Staphylococcus aureus*. Nat. Rev. Microbiol. 12, 49–62. doi: 10.1038/nrmicro3161, PMID: 24336184 PMC5708296

[ref21] Galperin M. Y. Wolf Y. I. Makarova K. S. Vera Alvarez R. Landsman D. Koonin E. V. (2021). COG database update: focus on microbial diversity, model organisms, and widespread pathogens. Nucleic Acids Res. 49, D274–D281. doi: 10.1093/nar/gkaa1018, PMID: 33167031 PMC7778934

[ref22] Gardner S. N. Slezak T. Hall B. G. (2015). kSNP3.0: SNP detection and phylogenetic analysis of genomes without genome alignment or reference genome. Bioinformatics 31, 2877–2878. doi: 10.1093/bioinformatics/btv271, PMID: 25913206

[ref23] Georgiades K. Raoult D. (2010). Defining pathogenic bacterial species in the genomic era. Front. Microbiol. 1:151. doi: 10.3389/fmicb.2010.00151, PMID: 21687765 PMC3109419

[ref24] Graham J. E. Moore J. E. Jiru X. Moore J. E. Goodall E. A. Dooley J. S.. (2007). Ocular pathogen or commensal: a PCR-based study of surface bacterial flora in normal and dry eyes. Invest. Ophthalmol. Vis. Sci. 48, 5616–5623. doi: 10.1167/iovs.07-0588, PMID: 18055811

[ref25] Gurevich A. Saveliev V. Vyahhi N. Tesler G. (2013). QUAST: quality assessment tool for genome assemblies. Bioinformatics 29, 1072–1075. doi: 10.1093/bioinformatics/btt086, PMID: 23422339 PMC3624806

[ref26] Hartman B. J. Tomasz A. (1984). Low-affinity penicillin-binding protein associated with beta-lactam resistance in *Staphylococcus aureus*. J. Bacteriol. 158, 513–516. doi: 10.1128/jb.158.2.513-516.1984, PMID: 6563036 PMC215458

[ref27] Henikoff S. Haughn G. W. Calvo J. M. Wallace J. C. (1988). A large family of bacterial activator proteins. Proc. Natl. Acad. Sci. U. S. A. 85, 6602–6606. 3413113 10.1073/pnas.85.18.6602 PMC282025

[ref28] Huerta-Cepas J. Forslund K. Coelho L. P. Szklarczyk D. Jensen L. J. Von Mering C.. (2017). Fast genome-wide functional annotation through orthology assignment by eggNOG-mapper. Mol. Biol. Evol. 34, 2115–2122. doi: 10.1093/molbev/msx148, PMID: 28460117 PMC5850834

[ref29] Huerta-Cepas J. Szklarczyk D. Heller D. Hernandez-Plaza A. Forslund S. K. Cook H.. (2019). eggNOG 5.0: a hierarchical, functionally and phylogenetically annotated orthology resource based on 5090 organisms and 2502 viruses. Nucleic Acids Res. 47, D309–D314. doi: 10.1093/nar/gky1085, PMID: 30418610 PMC6324079

[ref30] Jackson R. W. Vinatzer B. Arnold D. L. Dorus S. Murillo J. (2011). The influence of the accessory genome on bacterial pathogen evolution. Mob. Genet. Elements 1, 55–65. doi: 10.4161/mge.1.1.16432, PMID: 22016845 PMC3190274

[ref31] Katayama Y. Ito T. Hiramatsu K. (2000). A new class of genetic element, *Staphylococcus* cassette chromosome *mec*, encodes methicillin resistance in *Staphylococcus aureus*. Antimicrob. Agents Chemother. 44, 1549–1555. doi: 10.1128/AAC.44.6.1549-1555.2000, PMID: 10817707 PMC89911

[ref32] Keay L. Edwards K. Naduvilath T. Taylor H. R. Snibson G. R. Forde K.. (2006). Microbial keratitis predisposing factors and morbidity. Ophthalmology 113, 109–116. doi: 10.1016/j.ophtha.2005.08.013, PMID: 16360210

[ref33] Kirstahler P. Bjerrum S. S. Friis-Moller A. La Cour M. Aarestrup F. M. Westh H.. (2018). Genomics-based identification of microorganisms in human ocular body fluid. Sci. Rep. 8:4126. doi: 10.1038/s41598-018-22416-4, PMID: 29515160 PMC5841358

[ref34] Kleinschmidt S. Huygens F. Faoagali J. Rathnayake I. U. Hafner L. M. (2015). *Staphylococcus epidermidis* as a cause of bacteremia. Future Microbiol. 10, 1859–1879. doi: 10.2217/fmb.15.98, PMID: 26517189

[ref35] Koren S. Walenz B. P. Berlin K. Miller J. R. Bergman N. H. Phillippy A. M. (2017). Canu: scalable and accurate long-read assembly via adaptive k-mer weighting and repeat separation. Genome Res. 27, 722–736. doi: 10.1101/gr.215087.116, PMID: 28298431 PMC5411767

[ref36] Krzywinski M. Schein J. Birol I. Connors J. Gascoyne R. Horsman D.. (2009). Circos: an information aesthetic for comparative genomics. Genome Res. 19, 1639–1645. doi: 10.1101/gr.092759.109, PMID: 19541911 PMC2752132

[ref37] Larsen M. V. Cosentino S. Rasmussen S. Friis C. Hasman H. Marvig R. L.. (2012). Multilocus sequence typing of total-genome-sequenced bacteria. J. Clin. Microbiol. 50, 1355–1361. doi: 10.1128/JCM.06094-11, PMID: 22238442 PMC3318499

[ref38] Lee J. Y. H. Monk I. R. Goncalves Da Silva A. Seemann T. Chua K. Y. L. Kearns A.. (2018). Global spread of three multidrug-resistant lineages of *Staphylococcus epidermidis*. Nat. Microbiol. 3, 1175–1185. doi: 10.1038/s41564-018-0230-7, PMID: 30177740 PMC6660648

[ref39] Letunic I. Bork P. (2021). Interactive tree Of life (iTOL) v5: an online tool for phylogenetic tree display and annotation. Nucleic Acids Res. 49, W293–W296. doi: 10.1093/nar/gkab301, PMID: 33885785 PMC8265157

[ref40] Lichtinger A. Yeung S. N. Kim P. Amiran M. D. Iovieno A. Elbaz U.. (2012). Shifting trends in bacterial keratitis in Toronto: an 11-year review. Ophthalmology 119, 1785–1790. doi: 10.1016/j.ophtha.2012.03.031, PMID: 22627118

[ref41] Linehan J. L. Harrison O. J. Han S. J. Byrd A. L. Vujkovic-Cvijin I. Villarino A. V.. (2018). Non-classical immunity controls microbiota impact on skin immunity and tissue repair. Cell 172, 784.e718–796.e718. doi: 10.1016/j.cell.2017.12.033, PMID: 29358051 PMC6034182

[ref42] Liu B. Zheng D. Jin Q. Chen L. Yang J. (2019). VFDB 2019: a comparative pathogenomic platform with an interactive web interface. Nucleic Acids Res. 47, D687–D692. doi: 10.1093/nar/gky1080, PMID: 30395255 PMC6324032

[ref43] Manni M. Berkeley M. R. Seppey M. Simao F. A. Zdobnov E. M. (2021). BUSCO update: novel and streamlined workflows along with broader and deeper phylogenetic coverage for scoring of eukaryotic, prokaryotic, and viral genomes. Mol. Biol. Evol. 38, 4647–4654. doi: 10.1093/molbev/msab199, PMID: 34320186 PMC8476166

[ref44] McManus B. A. Coleman D. C. Deasy E. C. Brennan G. I. O’Connell B. Monecke S.. (2015). Comparative genotypes, staphylococcal cassette chromosome mec (SCCmec) genes and antimicrobial resistance amongst *Staphylococcus epidermidis* and *Staphylococcus haemolyticus* isolates from infections in humans and companion animals. PLoS One 10:e0138079. doi: 10.1371/journal.pone.0138079, PMID: 26379051 PMC4574763

[ref45] Nourdin-Galindo G. Sanchez P. Molina C. F. Espinoza-Rojas D. A. Oliver C. Ruiz P.. (2017). Comparative pan-genome analysis of piscirickettsia salmonis reveals genomic divergences within genogroups. Front. Cell. Infect. Microbiol. 7:459. doi: 10.3389/fcimb.2017.00459, PMID: 29164068 PMC5671498

[ref46] Oh J. Byrd A. L. Park M. Program N. C. S. Kong H. H. Segre J. A. (2016). Temporal stability of the human skin microbiome. Cell 165, 854–866. doi: 10.1016/j.cell.2016.04.008, PMID: 27153496 PMC4860256

[ref47] Otto M. (2004). Virulence factors of the coagulase-negative staphylococci. Front. Biosci. 9, 841–863. doi: 10.2741/1295, PMID: 14766414

[ref48] Otto M. (2009). *Staphylococcus epidermidis*–the ‘accidental’ pathogen. Nat. Rev. Microbiol. 7, 555–567. doi: 10.1038/nrmicro2182, PMID: 19609257 PMC2807625

[ref49] Park J. Y. Kim J. W. Moon B. Y. Lee J. Fortin Y. J. Austin F. W.. (2015a). Characterization of a novel two-component regulatory system, HptRS, the regulator for the hexose phosphate transport system in *Staphylococcus aureus*. Infect. Immun. 83, 1620–1628. doi: 10.1128/IAI.03109-14, PMID: 25644013 PMC4363416

[ref50] Park Y. M. Kwon H. J. Lee J. S. (2015b). Microbiological study of therapeutic soft contact lenses used in the treatment of recurrent corneal erosion syndrome. Eye Contact Lens 41, 84–86. doi: 10.1097/ICL.0000000000000068, PMID: 25230080

[ref51] Parks D. H. Imelfort M. Skennerton C. T. Hugenholtz P. Tyson G. W. (2015). CheckM: assessing the quality of microbial genomes recovered from isolates, single cells, and metagenomes. Genome Res. 25, 1043–1055. doi: 10.1101/gr.186072.114, PMID: 25977477 PMC4484387

[ref52] Piette A. Verschraegen G. (2009). Role of coagulase-negative staphylococci in human disease. Vet. Microbiol. 134, 45–54. doi: 10.1016/j.vetmic.2008.09.009, PMID: 18986783

[ref53] Qin L. Mccausland J. W. Cheung G. Y. Otto M. (2016). PSM-Mec-A virulence determinant that connects transcriptional regulation, virulence, and antibiotic resistance in staphylococci. Front. Microbiol. 7:1293. doi: 10.3389/fmicb.2016.01293, PMID: 27597849 PMC4992726

[ref54] Robert X. Gouet P. (2014). Deciphering key features in protein structures with the new ENDscript server. Nucleic Acids Res. 42, W320–W324. doi: 10.1093/nar/gku316, PMID: 24753421 PMC4086106

[ref55] Sayers E. W. Cavanaugh M. Clark K. Ostell J. Pruitt K. D. Karsch-Mizrachi I. (2020). GenBank. Nucleic Acids Res. 48, D84–D86. doi: 10.1093/nar/gkz956, PMID: 31665464 PMC7145611

[ref56] Schimel A. M. Miller D. Flynn H. W. Jr. (2013). Endophthalmitis isolates and antibiotic susceptibilities: a 10-year review of culture-proven cases. Am J. Ophthalmol. 156, 50.e51–52.e51. doi: 10.1016/j.ajo.2013.01.027, PMID: 23540710

[ref57] Schoenfelder S. M. Lange C. Eckart M. Hennig S. Kozytska S. Ziebuhr W. (2010). Success through diversity – how *Staphylococcus epidermidis* establishes as a nosocomial pathogen. Int. J. Med. Microbiol. 300, 380–386. doi: 10.1016/j.ijmm.2010.04.011, PMID: 20451447

[ref58] Schommer N. N. Gallo R. L. (2013). Structure and function of the human skin microbiome. Trends Microbiol. 21, 660–668. doi: 10.1016/j.tim.2013.10.001, PMID: 24238601 PMC4744460

[ref59] Seemann T. (2014). Prokka: rapid prokaryotic genome annotation. Bioinformatics 30, 2068–2069. doi: 10.1093/bioinformatics/btu153, PMID: 24642063

[ref60] Sharma S. Chaudhry V. Kumar S. Patil P. B. (2018). Phylogenomic based comparative studies on Indian and American commensal *Staphylococcus epidermidis* isolates. Front. Microbiol. 9:333. doi: 10.3389/fmicb.2018.00333, PMID: 29535698 PMC5835047

[ref61] Sievers F. Wilm A. Dineen D. Gibson T. J. Karplus K. Li W.. (2011). Fast, scalable generation of high-quality protein multiple sequence alignments using Clustal omega. Mol. Syst. Biol. 7:539. doi: 10.1038/msb.2011.75, PMID: 21988835 PMC3261699

[ref62] Stamatakis A. (2014). RAxML version 8: a tool for phylogenetic analysis and post-analysis of large phylogenies. Bioinformatics 30, 1312–1313. doi: 10.1093/bioinformatics/btu033, PMID: 24451623 PMC3998144

[ref63] Su F. Tian R. Yang Y. Li H. Sun G. Li Y.. (2020). Comparative genome analysis reveals the molecular basis of niche adaptation of *Staphylococcus epidermidis* strains. Front. Genet. 11:566080. doi: 10.3389/fgene.2020.566080, PMID: 33240320 PMC7680996

[ref64] Sun Z. Zhou D. Zhang X. Li Q. Lin H. Lu W.. (2020). Determining the genetic characteristics of resistance and virulence of the “Epidermidis Cluster Group” through pan-genome analysis. Front. Cell. Infect. Microbiol. 10:274. doi: 10.3389/fcimb.2020.00274, PMID: 32596166 PMC7303328

[ref65] Tettelin H. Masignani V. Cieslewicz M. J. Donati C. Medini D. Ward N. L.. (2005). Genome analysis of multiple pathogenic isolates of *Streptococcus agalactiae*: implications for the microbial “pan-genome”. Proc. Natl. Acad. Sci. U. S. A. 102, 13950–13955. doi: 10.1073/pnas.0506758102, PMID: 16172379 PMC1216834

[ref66] Vazquez V. Liang X. Horndahl J. K. Ganesh V. K. Smeds E. Foster T. J.. (2011). Fibrinogen is a ligand for the *Staphylococcus aureus* microbial surface components recognizing adhesive matrix molecules (MSCRAMM) bone sialoprotein-binding protein (Bbp). J. Biol. Chem. 286, 29797–29805. doi: 10.1074/jbc.M110.214981, PMID: 21642438 PMC3191021

[ref67] Willcox M. D. (2013). Characterization of the normal microbiota of the ocular surface. Exp. Eye Res. 117, 99–105. doi: 10.1016/j.exer.2013.06.003, PMID: 23797046

[ref68] Wisplinghoff H. Bischoff T. Tallent S. M. Seifert H. Wenzel R. P. Edmond M. B. (2004). Nosocomial bloodstream infections in US hospitals: analysis of 24,179 cases from a prospective nationwide surveillance study. Clin. Infect. Dis. 39, 309–317. doi: 10.1086/421946, PMID: 15306996

[ref69] Wos-Oxley M. L. Plumeier I. Von Eiff C. Taudien S. Platzer M. Vilchez-Vargas R.. (2010). A poke into the diversity and associations within human anterior nare microbial communities. ISME J. 4, 839–851. doi: 10.1038/ismej.2010.15, PMID: 20182526

[ref70] Zankari E. Hasman H. Cosentino S. Vestergaard M. Rasmussen S. Lund O.. (2012). Identification of acquired antimicrobial resistance genes. J. Antimicrob. Chemother. 67, 2640–2644. doi: 10.1093/jac/dks261, PMID: 22782487 PMC3468078

[ref71] Zhang Y. Liu Z. R. Chen H. Fan Y. C. Duo J. Zheng H.. (2013). Comparison on conjunctival sac bacterial flora of the seniors with dry eye in Ganzi autonomous prefecture. Int. J. Ophthalmol. 6, 452–457. doi: 10.3980/j.issn.2222-3959.2013.04.08, PMID: 23991377 PMC3755302

[ref72] Zhou W. Spoto M. Hardy R. Guan C. Fleming E. Larson P. J.. (2020). Host-specific evolutionary and transmission dynamics shape the functional diversification of *Staphylococcus epidermidis* in human skin. Cell 180, 454.e418–470.e418. doi: 10.1016/j.cell.2020.01.006, PMID: 32004459 PMC7192218

[ref73] Ziebuhr W. Hennig S. Eckart M. Kranzler H. Batzilla C. Kozitskaya S. (2006). Nosocomial infections by *Staphylococcus epidermidis*: how a commensal bacterium turns into a pathogen. Int. J. Antimicrob. Agents 28(Suppl 1), S14–S20. doi: 10.1016/j.ijantimicag.2006.05.012, PMID: 16829054

