# Supplementary material for: Comparative Genomic and Pan-Genomic Characterization of Staphylococcus epidermidis From Different Sources Unveils the Molecular Basis and Potential Biomarkers of Pathogenic Strains
Source: Front Microbiol. 2021 Nov 15;12:770191. doi: 10.3389/fmicb.2021.770191 (PMC8634615; doi:10.3389/fmicb.2021.770191)
Supplement: Supplementary file 1 [file Table_1.DOCX]

Supplementary Material

# Supplementary Table

**Supplementary Table1|**The Source information of 176 downloaded *S. epidermidis* sequences

| **Strain** | **Isolation source** | **Host healthy state** | **Accession number** |
| --- | --- | --- | --- |
| SESURV_p3_1362 | L_index( skin of manus ) | Healthy | PRJNA559376 |
| SESURV_p1_1200 | Cubital_fossa(arm skin) | Healthy | PRJNA559376 |
| SESURV_p3_0825 | L_thumb(skin of manus) | Healthy | PRJNA559376 |
| SESURV_p2_0614 | R_thumb( skin of manus ) | Healthy | PRJNA559376 |
| SESURV_p1_0563 | R_cheek(skin of face ) | Healthy | PRJNA559376 |
| SESURV_p1_0557 | R_nares(external naris) | Healthy | PRJNA559376 |
| SESURV_p0_312 | Forehead(skin of face) | Healthy | PRJNA559376 |
| AMT | skin and mucous membranes | Unsure | PRJNA392321 |
| VSE43 | Skin | Healthy | PRJNA348420 |
| VSE44 | Skin | Healthy | PRJNA348422 |
| VSE46 | Skin | Healthy | PRJNA348426 |
| VSE47 | Skin | Healthy | PRJNA348434 |
| VSE48 | Skin | Healthy | PRJNA348435 |
| VSE49 | Skin | Healthy | PRJNA348436 |
| VSE54 | Skin | Healthy | PRJNA348442 |
| VSE56 | Skin | Healthy | PRJNA348443 |
| VSE58 | Skin | Healthy | PRJNA348446 |
| VSE37 | Skin | Healthy | PRJNA348414 |
| VSE39 | Skin | Healthy | PRJNA348415 |
| VSE41 | Skin | Healthy | PRJNA348416 |
| VSE3 | Skin | Healthy | PRJNA291322 |
| SE35 | Behind the ears | Healthy | PRJNA314011 |
| SE42 | Behind the ears | Healthy | PRJNA314011 |
| SE47 | Behind the ears | Healthy | PRJNA314011 |
| SE49 | Behind the ears | Healthy | PRJNA314011 |
| SE56 | Behind the ears | Healthy | PRJNA314011 |
| SE57 | Behind the ears | Healthy | PRJNA314011 |
| SE43 | Ear | Healthy | PRJNA314011 |
| SE39 | Nares | Healthy | PRJNA314011 |
| SE40 | Nares | Healthy | PRJNA314011 |
| SE48 | Nares | Healthy | PRJNA314011 |
| SE52 | Nares | Healthy | PRJNA314011 |
| SE53 | Nares | Healthy | PRJNA314011 |
| CDC120 | Skin | Unsure | PRJNA506796 |
| CDC121 | Skin | Unsure | PRJNA506797 |
| HD33 | Skin | Prosthesis Related Infections | PRJNA546129 |
| HD43 | Skin | Prosthesis Related Infections | PRJNA546129 |
| HD66 | Skin | Prosthesis Related Infections | PRJNA546129 |
| O47 | Skin | Unsure | PRJNA546513 |
| ST14 | Skin | Unsure | PRJNA289935 |
| SK135 | Skin | Unsure | PRJNA34077 |
| MO34 | Forearm | Unsure | PRJDB6506 |
| MFP04 | cheekbone | Unsure | PRJNA649214 |
| NIHLM001 | alar crease | Unsure | PRJNA62387 |
| NIHLM008 | alar crease | Unsure | PRJNA62383 |
| NIHLM070 | alar crease | Unsure | PRJNA62351 |
| NIHLM061 | Nares | Unsure | PRJNA62355 |
| NIHLM087 | Nares | Unsure | PRJNA62349 |
| NIHLM088 | Nares | Unsure | PRJNA62347 |
| MTCC 3382 | Nose | Unsure | PRJNA293017 |
| NCTC6513 | Nose | Unsure | PRJEB6403 |
| NCTC10519 | Nasal swab | Unsure | PRJEB6403 |
| CV94 | Nasal swab | Unsure | PRJNA308322 |
| HESS022 | Nasal mucosa swab | Colonization | PRJNA668279 |
| HESN035b | Nasal mucosa swab | Colonization | PRJNA668279 |
| NIHLM037 | Glabella | Unsure | PRJNA62369 |
| NIHLM053 | Gypothenar palm | Unsure | PRJNA62359 |
| AK-612 | Ear | Ear infection | PRJNA525578 |
| IRL01 | Blood | Staphylococcal Infection | PRJNA532483 |
| NCCP 16828 | Blood | Diabetes, Hypertension | PRJNA564725 |
| DAR1907 | Blood | Diseased | PRJNA308322 |
| DAR5907 | Blood | Diseased | PRJNA308322 |
| DAR5908 | Blood | Diseased | PRJNA308322 |
| DAR5909 | Blood | Diseased | PRJNA308322 |
| DAR5153 | Blood | Diseased | PRJNA308322 |
| DAR5988 | Blood | Diseased | PRJNA308322 |
| DAR5954 | Blood | Diseased | PRJNA308322 |
| NCTC13924 | Blood culture | Diseased | PRJEB6403 |
| SE90 | Blood cultures | Bacteremia | PRJNA412200 |
| SE95 | Blood cultures | Bacteremia | PRJNA412208 |
| 8400 | Blood cultures | Diseased | PRJNA238030 |
| 1457 | Central veneous catheter | Diseased | PRJNA238030 |
| 1057 | Central veneous catheter | Diseased | PRJNA238030 |
| 94 | Blood cultures | Blood Infection | PRJNA504381 |
| 23 | Blood cultures | Blood Infection | PRJNA504381 |
| GPR20 | Blood cultures | Blood Infection | PRJNA504381 |
| FDAARGOS_161 | Peripheral blood | Diseased | PRJNA231221 |
| FDAARGOS_153 | Peripheral blood | Diseased | PRJNA231221 |
| FDAARGOS_157 | Peripheral blood | Diseased | PRJNA231221 |
| FDAARGOS_529 | CVP line blood | Diseased | PRJNA231221 |
| SH03_17 | Blood | Bacteremia | PRJNA419705 |
| SH06_17 | Blood | Bacteremia | PRJNA419711 |
| SH07_17 | Blood | Bacteremia | PRJNA419710 |
| SH06_16 | Blood | Bacteremia | PRJNA419706 |
| NIH04003 | Blood | Diseased | PRJNA86759 |
| NIH05003 | Blood | Diseased | PRJNA86761 |
| NIH06004 | Blood | Diseased | PRJNA86763 |
| NIH08001 | Blood | Diseased | PRJNA86765 |
| NGS-ED-1107 | Blood | Blood Sepsis | PRJNA255947 |
| NGS-ED-1109 | Blood | Blood Sepsis | PRJNA255947 |
| NGS-ED-1110 | Blood | Blood Sepsis | PRJNA255947 |
| NGS-ED-1111 | Blood | Blood Sepsis | PRJNA255947 |
| NGS-ED-1118 | Blood | Blood Sepsis | PRJNA255947 |
| S12 | Blood | Nosocomial | PRJNA526018 |
| S09 | Blood | Nosocomial | PRJNA526018 |
| S21 | Catheter from newborn | Nosocomial | PRJNA526018 |
| S03 | Catheter from newborn | Nosocomial | PRJNA526018 |
| S19 | Catheter from newborn | Nosocomial | PRJNA526018 |
| HESN074B | Blood | Diseased | PRJNA668279 |
| HESN016B | Blood | Diseased | PRJNA668279 |
| hHESN103B | Blood | Diseased | PRJNA668279 |
| HESN038B | Blood | Diseased | PRJNA668279 |
| HESN090B | Blood | Diseased | PRJNA668279 |
| C133 | Blood | Diseased | PRJNA667485 |
| C138 | Blood | Diseased | PRJNA667485 |
| MEX86 | Blood | Diseased | PRJNA308322 |
| MCO7 | Blood | Diseased | PRJNA308322 |
| 12142587 | Blood cultures | community-acquired native valve endocarditis | PRJNA175894 |
| AUH4567 | Blood and central venous catheter | Central venous catheter infection | PRJNA374795 |
| NCTC11964 | Prosthetic heart valve | Diseased | PRJEB6403 |
| AU12-03 | Intravascular catheter | Diseased | PRJNA171207 |
| I6-23.3 | Human eye | Diseased | PRJEB21503 |
| I6-23.2 | Human eye | Diseased | PRJEB21503 |
| I6-23.1 | Human eye | Diseased | PRJEB21503 |
| C7 | Human eye | Diseased | PRJEB21503 |
| I4_5 | Human eye | Diseased | PRJEB21503 |
| C3 | Human eye | Diseased | PRJEB21503 |
| MRSE 52-2 | Pharyngeal exudade | Healthy | PRJNA402084 |
| HUR103 | Respiratory | Unsure | PRJNA308322 |
| HUR104 | Respiratory | Unsure | PRJNA308322 |
| ICE25 | Respiratory | Unsure | PRJNA308322 |
| NIH051668 | Lung | Unsure | PRJNA86757 |
| LRKNS083 | Bronchoalveolar lavage | Colonisation | PRJNA314440 |
| ET-024 | Endotracheal tube biofilm of a mechanically ventilated patient | Diseased | PRJNA241149 |
| C10C | Sputum | Unsure | PRJNA246628 |
| SAM-3 | Sputum | Unsure | PRJNA437720 |
| 0514Y_32_9 | Oral | Unsure | PRJNA327106 |
| Se_BPH0711 | Clinical | Diseased | PRJEB35032 |
| Se_BPH0697 | Clinical | Diseased | PRJEB35032 |
| Se_RP62a-WT | Clinical | Diseased | PRJEB35032 |
| Se_BPH0723 | Clinical | Diseased | PRJEB35032 |
| Se_BPH0736 | Clinical | Diseased | PRJEB35032 |
| Se_BPH0704 | Clinical | Diseased | PRJEB35032 |
| SEI(ATCC 49134) | Clinical isolate | Unsure | PRJNA244351 |
| B647 | Clinic | Colonisation | PRJNA319642 |
| LRKNS062 | Clinic | Colonisation | PRJNA314440 |
| LRKNS064 | Clinic | Colonisation | PRJNA314440 |
| LRKNS076 | Clinic | Colonisation | PRJNA314440 |
| LRKNS077 | Clinic | Colonisation | PRJNA314440 |
| LRKNS020 | Clinic | Colonisation | PRJNA314440 |
| LRKNS037 | Clinic | Colonisation | PRJNA314440 |
| LRKNS039 | Clinic | Colonisation | PRJNA314440 |
| LRKNS048 | Clinic | Colonisation | PRJNA314440 |
| LRKNS056 | Clinic | Colonisation | PRJNA314440 |
| LRKNS057 | Clinic | Colonisation | PRJNA314440 |
| LRKNS113 | Clinic | Colonisation | PRJNA314440 |
| LRKNS114 | Clinic | Colonisation | PRJNA314440 |
| LRKNS112 | Clinic | Colonisation | PRJNA314440 |
| LRKNS116 | Clinic | Colonisation | PRJNA314440 |
| LRKNS017 | Clinic | Colonisation | PRJNA314440 |
| LRKNS029 | Clinic | Colonisation | PRJNA314440 |
| LRKNS054 | Clinic | Colonisation | PRJNA314440 |
| LRKNS059 | Clinic | Colonisation | PRJNA314440 |
| LRKNS072 | Clinic | Colonisation | PRJNA314440 |
| AOKB02 | Clinic | Colonisation | PRJNA319644 |
| ALKB06 | Clinic | Colonisation | PRJNA319642 |
| A1KF08 | Clinic | Colonisation | PRJNA319644 |
| AHKF08 | Clinic | Colonisation | PRJNA319645 |
| ACEF09 | Clinic | Colonisation | PRJNA319642 |
| AEEG06 | Clinic | Colonisation | PRJNA319645 |
| Bt1p3 | Clinic | Colonisation | PRJNA319644 |
| Qt1p44 | Clinic | Colonisation | PRJNA319644 |
| pK_B01 | Clinic | Colonisation | PRJNA319642 |
| B59 | Clinic | Colonisation | PRJNA319642 |
| B7234 | Clinic | Colonisation | PRJNA319642 |
| B13258 | Clinic | Colonisation | PRJNA319642 |
| B13587 | Clinic | Colonisation | PRJNA319642 |
| B2731 | Clinic | Colonisation | PRJNA319642 |
| B10873 | Clinic | Colonisation | PRJNA319642 |
| B12916 | Clinic | Colonisation | PRJNA319642 |
| B12142 | Clinic | Colonisation | PRJNA319642 |
| KED12 | Clinic | Colonisation | PRJNA319644 |
| Kt1p58 | Clinic | Colonisation | PRJNA319642 |
| RP62A | Reference strains | Unsure | PRJNA64 |
| ATCC 14990 | Reference strains | Unsure | PRJNA515334 |
| ATCC 12228 | Reference strains | Unsure | PRJNA564724 |

|  | **Mapping Reads** | **Unmappin-g Reads** | **Mean Coverage Depth** | **Coverage Depth<10（%）** | **GC content** | **Contig number** | **Genome size（Mb）** | **CDS** | **tRNA** | **rRNA** |
| --- | --- | --- | --- | --- | --- | --- | --- | --- | --- | --- |
| O106 | 1063834 | 0 | 4524 | <0.001% | 32.27% | 2 | 2.54 | 2360 | 60 | 19 |
| O107 | 1635910 | 0 | 6830 | 0 | 32.15% | 3 | 2.56 | 2355 | 60 | 19 |
| O108 | 1148008 | 0 | 5347 | 0 | 32.24% | 1 | 2.49 | 2247 | 60 | 22 |
| O109 | 1457938 | 0 | 6404 | 0.0023% | 32.10% | 3 | 2.56 | 2391 | 59 | 19 |
| O110 | 1523429 | 0 | 6511 | 0 | 32.05% | 3 | 2.67 | 2446 | 60 | 19 |
| O112 | 1629802 | 0 | 6936 | 0 | 32.17% | 2 | 2.54 | 2313 | 59 | 19 |
| O113 | 1495273 | 0 | 6420 | 0.016% | 32.04% | 4 | 2.62 | 2455 | 60 | 19 |
| O114 | 1485509 | 0 | 6590 | <0.001% | 32.15% | 4 | 2.58 | 2345 | 60 | 19 |
| O115 | 988780 | 0 | 4438 | 0 | 32.15% | 2 | 2.51 | 2272 | 60 | 19 |
| O116 | 1110019 | 0 | 4641 | 0.1% | 32.01% | 6 | 2.79 | 2692 | 60 | 19 |
| O117 | 1130833 | 0 | 5414 | <0.001 | 32.27% | 1 | 2.48 | 2265 | 61 | 19 |

**Supplementary Table2|**Genomic characteristics of 11 ocular trauma-source *Staphylococcus epidermidis*
